# Dialogic scientific gatherings: promoting inclusive health participation and communication among non-academic Roma women

**DOI:** 10.3389/fpubh.2025.1618150

**Published:** 2025-07-10

**Authors:** Ariadna Munté-Pascual, Laura Ruiz-Eugenio, Ane López de Aguileta

**Affiliations:** ^1^Social Work Training and Research Section, University of Barcelona, Barcelona, Spain; ^2^Department of Theory and History of Education, University of Barcelona, Barcelona, Spain

**Keywords:** health communication, vulnerable groups, Roma women, community intervention, dialogic approach

## Abstract

There is evidence that Dialogic Scientific Gatherings (DSG) are an effective intervention for inclusive health communication, demonstrating a positive impact on promoting healthy habits. This is particularly relevant for groups that typically face more health issues due to socioeconomic and educational inequalities. However, the implementation of DSG with non-academic Roma women, one of the groups historically suffering from these inequalities, has not yet been studied. To address this knowledge gap, within the framework of the ROM21 “Roma Women Leading Communities’ Transformation” research project, four DSG sessions were co-created with non-academic Roma women, in collaboration with a Roma women’s association and the research team. The DSG sessions focused on health topics chosen by the participants themselves. The analysis of the interventions by Roma women in the DSG revealed that these sessions provided a space for the collective creation of meaning and knowledge on health topics that concern them. This contributed to overcoming stereotypes about their lack of interest in evidence-based health information that promotes healthy habits. Topics discussed included the relationship between sedentary behavior and diet with diseases such as cancer and childhood obesity, as well as the connection between social ties and mental health. These discussions facilitated an environment conducive to informed health decisions among non-academic Roma women.

## 1 Introduction

Roma women play a crucial role in promoting health equity and reducing health disparities within their communities by actively participating in health education, advocacy, and community-based interventions (1). These efforts are essential in addressing the unique health challenges faced by Roma communities, which are often characterized by poor health literacy, limited access to healthcare, and significant health inequalities compared to non-Roma populations (2). Roma women, through various initiatives, have become pivotal in driving change and improving health outcomes in their communities (3, 4). Qualitative study in Spain demonstrates how Roma women have led initiatives to preserve the health of their communities during the COVID-19 pandemic, challenging the negative stereotypes often attributed to them, and underscoring the importance of collaboration between Roma women and health and social service organizations to address health inequalities (3).

Roma women have played a crucial role in tailored training programs that emphasize health and prevention topics, including gynecological health, vaccinations, smoking cessation, and nutrition. These initiatives are designed to enhance health literacy and empower Roma women to make informed health decisions (5). Furthermore, Roma women have been central to community-based participatory action research, which seeks to address health disparities by engaging local stakeholders, such as healthcare providers and community organizations, in the formulation of health equity agendas (6).

While Roma women have made significant strides in promoting health equity, challenges remain. The health disparities affecting Roma communities are deeply rooted in social and economic inequities, and systemic discrimination continues to hinder progress (7). To further advance this challenge, within the framework of one of the case studies of the ROM21 “Roma women leading communities’ transformation” research project, four Dialogic Scientific Gatherings (DSG) sessions were developed with non-academic Roma women, in collaboration with a Roma women’s association and the research team. DSG is an evidence-based intervention of its effectiveness in promoting informed decisions based on reading and dialog about scientific health evidence with vulnerable groups (8, 9). However, this intervention had never been carried out with non-academic Roma women. The research question addressed by the presented case study is: What impact have DSG sessions had in promoting an environment conducive to informed health decision-making among the participating Roma women?

## 2 The health communication approach in this case study

This study adopts a critical, dialogic perspective on health communication, viewing it not merely as a unidirectional channel for transmitting information, but as a dialogic process that shapes how individuals access, interpret, and apply health-related knowledge through collective meaning-making (10, 11). This model emphasizes reciprocal, horizontal communication that fosters mutual understanding, co-creation of knowledge, and community empowerment (12, 13). It draws on Paulo Freire’s pedagogical principles and aligns with health promotion strategies that value local knowledge and collective agency (14). Our approach is consistent with contemporary frameworks that position health communication as a tool for advancing equity, social justice, and the transformation of the social determinants of health (14–16).

Within this framework, we incorporate contributions to health risk communication with vulnerable populations (17). These contributions highlight the need for clear, culturally responsive, and motivating communication strategies to address health disparities affecting underserved groups—such as individuals with low health literacy, cultural barriers, or economic constraints (18).

This perspective considers not only the content of communication but also the relational dynamics that emerge in the process. Establishing a dialogic space for health communication within vulnerable communities involves creating environments where all voices are heard and valued based on the strength of their arguments rather than hierarchical status. Such spaces are grounded in the collective construction of knowledge, where scientific and experiential insights converge to inform more inclusive and effective health practices. In this context, communicative acts are analyzed not only for their explicit content but also for their potential to transform relationships, build trust, and foster active participation in decisions affecting individual and collective well-being (13, 19).

This approach is rooted in the principle that all individuals, regardless of educational background or social position, can contribute valid arguments to shared knowledge construction (9). Dialogic spaces thus go beyond the transmission of scientific information to promote mutual understanding, empowerment, and shared responsibility in health care. Interaction becomes a transformative communicative act, where language not only reflects reality but actively reshapes it through the engagement of all participants (19).

The model is inspired by the theory of the dialogic society, which posits that social transformation emerges through interactions grounded in knowledge co-creation and the strength of arguments rather than hierarchical authority (19). It also incorporates the analysis of communicative acts as a means to distinguish between those that reproduce power imbalances and those that foster dialogic relationships (20). In community settings, this translates into practices such as dialogic health gatherings, where participants engage with scientific texts on health and well-being, promoting critical and context-sensitive health literacy. These dialogic spaces become collective learning environments that recognize cultural diversity and promote equitable access to scientific knowledge. Within this framework, dialogic scientific gatherings (DSG) exemplify this approach by creating inclusive spaces for dialogic engagement with scientific evidence, enabling participants to collectively interpret knowledge and make informed decisions (8). Contextualization of the case study: the ROM21 research project

The ROM21 “Roma Women Leading Communities’ Transformation” research project has been funded by the Spanish Research Agency under the Ministry of Science, Innovation, and Universities. Its primary goal is to generate knowledge with both scientific and social impact, focusing on initiatives led by Roma women to overcome the inequalities faced by the Roma community in Spain.

The ROM21 project identified initiatives co-created with Roma women that promoted their leadership in overcoming inequalities within their communities. Among these initiatives, the approach of bringing scientific health evidence to Roma women as key socialization agents in their communities was highlighted. The creation of dialog spaces for access to evidence-based health information and mutual support networks, driven by the women themselves in collaboration with community organizations and social services, was crucial for Roma women to play an active role in health preservation during the COVID-19 pandemic. This included both physical health (prevention of contagion) and mental health (moral support, maintaining the mental health of families) (3).

Building on the prior knowledge generated in ROM21 and previous evidence on DSGs as an effective intervention for promoting informed health decisions, it was proposed to the Roma Women’s Association Drom Kotar Mestipen [Path to Freedom in Romani], with whom collaboration has been ongoing throughout the development of ROM21, to conduct DSGs on health topics chosen by the women themselves. This article presents the results of this case study, addressing the previously referenced research question.

## 3 Materials and methods

### 3.1 The intervention: dialogic scientific gatherings with Roma women

In Dialogic Gatherings, a collective construction of meaning and knowledge is produced based on the dialog of the participants through the best creations of humanity in various fields from literature, art, or science, among others (21). Dialogic Gatherings began in an adult school in Barcelona during the 1979–1980 academic year. Initially aimed at adults with basic educational levels, these gatherings have expanded to all ages and various contexts, from elementary schools to primary health care centers. In Dialogic Scientific Gatherings, participants read and discuss scientific knowledge or its applications, based on dialogic learning. Egalitarian dialog ensures that the value of contributions does not depend on hierarchies. Participants read the text beforehand and share their reflections. The moderator prioritizes the participation of those who have spoken less, creating a space for the collective creation of meaning and knowledge (8).

#### 3.1.1 The co-created proposal with non-academic Roma women for DSGs on health

Considering the previous findings of the ROM21 project (3), the researchers proposed to the Roma Women’s Association Drom Kotar Mestipen to conduct DSGs on health with the women associated with their organization who wished to participate. This association has been involved in the ROM21 research project throughout all its development phases, including its design. The association consists of a network of Roma women, most of whom have basic educational levels or no academic qualifications, spread across the Autonomous Community of Catalonia in Spain. In this study, the term ‘non-academic Roma women’ refers to women without higher education who are active members of the Roma Women’s Association Drom Kotar Mestipen. The association’s aim is to promote educational and social initiatives to overcome the inequalities faced by Roma women and girls, as well as their communities. The DSGs were co-organized with the association and the research team. The association facilitated recruitment, follow-up, and ongoing communication with participants through WhatsApp groups and regular meetings. Several women from the association expressed their interest in participating. The research team asked them about the topics they wanted to address in the DSGs, their availability, and their preference for the DSG format (in-person/online). Due to the COVID-19 lockdown, they had already become accustomed to the online format for some of the association’s activities. Initially, there was a preference for in-person meetings, but the supportive dynamic among the women in this association ultimately led to alternating between in-person and online formats. This approach facilitated the participation of women from geographically distant areas who had difficulties traveling to the association’s headquarters.

The topics they decided to address in the DSGs were: (1) habits and cancer prevention; (2) mental health; (3) childhood obesity; (4) pharmaceuticals and alternative remedies.

#### 3.1.2 Preparation for the DSG

The researchers searched for scientific articles on these topics in open-access, peer-reviewed journals indexed in major databases. The criteria for selecting the scientific articles were: (1) they should present contributions easily relatable to the daily lives of the women and their communities, and (2) they should provide elements that help reinforce or modify habits, as well as overcome conceptions not based on scientific evidence, with the intention of promoting individual and community health.

Once the articles were selected, those in English were automatically translated into Spanish using AI. Subsequently, the researchers synthesized the texts to reduce them to about 4 or 5 pages, considering that most participants have basic or no formal education. The paragraphs in the synthesis were literal from the original version. Special attention was given to not adulterate the content of the contributions. The synthesis included the full reference of the article and the DOI through which the original manuscript could be accessed. This synthesis was sent via email to the association, which then distributed it among the DSG participants either in paper format or through mobile messaging groups, according to each participant’s preference.

The participating women received the text several days before the DSG so they could read it in advance. During the preliminary reading, each participant chose at least one paragraph to highlight and share during the gathering. Participation was promoted through the association’s internal communication channels, including WhatsApp groups and word-of-mouth.

#### 3.1.3 Conducting the DSG

Each DSG session included between 6 and 9 participants, depending on availability. All four sessions were attended by a core group of women, with some variation in attendance. Four DSG sessions were conducted between January and May 2024, one for each of the topics chosen by the participating women (see Table 1). In-person sessions were held at the headquarters of the Roma Women’s Association in Barcelona, while online sessions were conducted via Zoom to facilitate participation from women in other regions.

**Table 1:** Papers read on each DSG session.

Dates	Format	Participants	Topic	Reference
11/01/2024	In-person	9	Physical activity, sedentary behavior, diet, and cancer	(22)
19/02/2024	Online	8	Social ties and mental health.	(23)
14/03/2024	In-person	9	Childhood obesity and guidelines for improving weight control	(24)
31/05/2024	Online	6	Fibromyalgia, recent advances in diagnosis, classification, pharmacotherapy and alternative remedies.	(25)

The DSGs were co-organized by the research team and the Roma Women’s Association. Researchers acted as facilitators and moderators, while the association coordinated logistics, participant outreach, and follow-up. The step-by-step process for conducting the DSG is explained below, which followed the same procedure for both in-person and online sessions. Each session lasted approximately 1.5 h.

##### 3.1.3.1 Step 1

The researcher situates the DSG within the framework of the ROM21 research project. The objective of the research is explained, highlighting how the proposal for this DSG emerged from what Roma individuals have shared about what helped them during the pandemic, such as having accurate and reliable health information to make informed decisions. It is communicated that the DSG will be transcribed anonymously for analysis and that the results will be used to develop scientific articles and communications aimed at ensuring that scientific evidence on health topics reaches everyone. Participants are informed that their participation is voluntary and that they can withdraw at any time without needing to provide any explanation. The contact information of the principal investigator is provided, and she also participates in the DSG, allowing the women to ask any questions they may have.

##### 3.1.3.2 Step 2

The concept of scientific journals was explained, including where and why the article was chosen, how the synthesis was created, and the possibility of accessing the original through the DOI link. Participants were asked if they wanted to re-read the synthesis at that moment or start the discussion directly. In all four DSG, they chose to re-read the text. This moment was used to address any questions that arose or clarify vocabulary, using accessible language while also helping to incorporate new vocabulary.

##### 3.1.3.3 Step 3

The fundamental dialogic principles for the functioning of the DSG were presented. Anyone who wanted to share the paragraph they had selected would request to speak from the moderator, who in this case was one of the researchers. The moderator would then give the floor and moderate the debate. To participate, one must request to speak and avoid interrupting others. All opinions were valued and respected based on the arguments they presented, without following any hierarchy (e.g., between researchers and participants). Egalitarian dialog involves respecting different contributions without imposing one’s own views. The moderator prioritized giving the floor to those who had not yet spoken or had spoken less. She could encourage participation by connecting different interventions and valuing all contributions, but without monopolizing the debate. Before concluding each session, a few minutes were dedicated for anyone who wished to provide oral feedback on their experience of the DSG. It is worth noting that spontaneous evaluative comments about the text and the usefulness of the DSG emerged throughout the session.

### 3.2 Data collection

The evidence collected comes from quotes extracted from the analysis of the literal transcriptions of the four DSG sessions. The key messages and reflections emerged organically during the discussions and were later synthesized by the research team through thematic analysis of the transcriptions. The challenge of identifying who was speaking in all the interventions when listening to the audio of the four DSG sessions led to not identifying which participant made each intervention, as this was not crucial for the analysis. Instead, the focus was on the collective creation of meaning and knowledge. Therefore, following dialogic principles, the most important aspect of the analysis was identifying the contributions and arguments that generated the most debate or achieved the greatest consensus. The analysis focused on identifying recurring themes, consensus points, and contrasting perspectives across sessions. While each session addressed a different topic, the analysis also explored transversal dynamics such as participation patterns, types of arguments, and expressions of mutual learning. All the participants contributed multiple times in every DSG session. Nine Romani women participated in the DSG, along with members of the research team (see Table 2). Among the nine participants, six had completed only primary education, one had finished compulsory secondary education, and the two women currently working as social worker and educator in the association where the DSG was organized hold university degrees.

**Table 2:** Profile of the Romani women participants in the DSG.

Code	Marital status	Employment status	Children	Age range	ISCED*
P1	Married	Temporary work	3	35–40	L1
P2	Single	Social Educator	–	30–34	L5
P3	Divorced	Social facilitator	–	35–40	L2
P4	Cohabiting partner	Sick leave	4	35–40	L1
P5	Separated	Unemployed	2	35–40	L1
P6	Cohabiting partner	Cleaner	2	35–40	L1
P7	Single	Social Worker	-	25–30	L5
P8	Married	Temporary work	3	45–50	L1
P9	Married	Temporary work	3	45–50	L1

*ISCED = International Standard Classification of Education (1997): NBS = No basic studies; L0 = Pre-primary education; L1 = Primary Education 1–6; L2 = Lower Secondary Education 1–4; L3 = Upper Secondary Education 1–2; L4 = Postsecondary nontertiary education; L5 = First stage of tertiary education 1–3/4; L6 = Second stage of tertiary education ½.

Based on previous evidence and the analysis of the transcriptions, the categories of analysis have been established (see Table 3).

**Table 3:** Categories of analysis.

Category	Subcategory
Knowledge acquisition	Known topics New information on health topics
Interest in the topics discussed	On the topic discussed On other health topics
Application of information	In themselves, their families, and communities

## 4 Results

### 4.1 Knowledge acquisition

#### 4.1.1 Pre-existing knowledge and reinforcement of acquired habits

The DSG sessions revealed that participants already possessed relevant health knowledge, which was reinforced through the reading and discussion of scientific articles. This validation process strengthened their confidence in their existing practices and encouraged further reflection. The discussions particularly highlighted the connection between mental health and social stressors, as well as the role of community support in emotional well-being.

During the four DSG sessions, the participants’ interventions highlighted how the articles confirmed and reinforced knowledge they already possessed through their own experiences or acquired habits. For instance, in the session on mental health, participants identified the social origins of anxiety and the tendency toward medicalization without addressing root causes. One participant mentioned that, as a Romani woman who has worked in her neighborhood with many women, she identified that many mental health issues, such as anxiety, were caused by a social context where circumstances accumulated due to the overload of responsibilities from balancing work, household chores, child-rearing, and caring for other dependent family members, compounded by financial worries and other concerns. This woman pointed out that, despite anxiety being a mental health issue with social origins, it is immediately medicalized without addressing the root of the problem. Other participants joined in with various interventions similar to the following to confirm this fact:

Imagine you go to the doctor with an anxiety attack... I’ve worked in the neighborhood as a mediator, and sometimes I accompany some women. So what happens is that most of the conflicts and problems they face come from the same thing: stress, not being able to keep up, all that together emotionally, they break down. So, what happens when they go to the doctor? The first thing they get is Diazepam

This insight was echoed by others, who emphasized the gendered burden of emotional care. Some interventions also identified how, in the daily lives of women in their community, being a woman can partly explain the higher prevalence of psychological distress compared to men, as described in the article. This is especially true when connected to concerns arising from work, family care, and household economy. One of the participants, supported by several interventions along the same lines, reflected on gender roles and the emotional burden assigned to women: “If we are the ones responsible for providing emotional support in the face of all the family’s difficulties, in the end, we are also the ones who are more exposed”.

The interventions of these participants demonstrate that they already understood the relationship between being a woman, the stress of daily life, exacerbated by various social issues, and mental health. Furthermore, they emphasized the importance of social support in addressing these problems rather than immediately resorting to medicalization as the sole response.

Participants also recognized the value of community involvement and social ties in promoting mental health. The participants identified the importance of community involvement, quality social bonds, and support networks in their daily lives as key elements for preventing and overcoming mental health issues. These women already understood this relationship through their own experiences, and the article they read and discussed in the gathering confirmed and reinforced this knowledge.

One woman linked her professional experience to the article’s findings. This woman connected her work with involvement in community organizations, highlighting how these activities strengthen social bonds and contribute to mental well-being. Several participants confirmed this fact with their own experiences: “I related it to what I do at the school where I work. I related it when it talks about participation in organizations or community organizations as preventive factors.” This idea was further developed through reflections on solidarity and mutual care. These interventions demonstrated an understanding that community participation can provide a sense of belonging and support, which is crucial for mental health. The article confirmed that such social bonds are essential for emotional well-being. One participant spoke about the spread of stress and the importance of solidarity among women. She emphasized the significance of sharing experiences to support each other, enabling women to relieve stress and improve their emotional well-being: “To prevent the spread of stress, regarding what you said about balance, I related it to how we, as women, share this and that, family situations, things….”

Another participant also highlighted participation in volunteer activities as a way to improve symptoms of depression. Solidarity and support within the community, especially among Romani women, have been fundamental to their well-being:

I also found it important (in the article) that participation in volunteer activities alleviated or improved women's depression symptoms. Solidarity and helping each other among Romani women happens and has always happened. Moreover, we are now seeing more involvement, with them participating in school and other places.

The article reaffirmed that these practices of mutual support and community participation are effective in improving mental health. Finally, one of the participants reflected on how what they had already identified as influencing their mental health negatively, such as socioeconomic difficulties, and positively, such as community participation, was confirmed by the evidence provided in the article, which they can now use to strengthen their arguments:

What we've been discussing about participation and how some issues like socioeconomic factors influence it, well, it's true that in the end, it's not just things you think about, but you know there are studies that support it. When we talk about it, we'll have more arguments to provide some explanations.

These quotes reflect a deep understanding of how social bonds and community participation not only prevent mental health issues but also offer a path to recovery and well-being. The article they read and discussed in that DSG session provided additional validation that their experiences and habits are supported by studies and scientific evidence.

Another topic that generated numerous interventions, confirming not only the participants’ prior knowledge but also reaffirming the changes in habits they had already adopted, was the DSG session on physical activity, sedentary lifestyle, diet, and cancer. There were several interventions that corroborated their prior understanding of the importance of physical exercise and nutrition in preventing not only obesity but also other diseases, as well as the reaffirmation of habits that some participants had already adopted:

(The article) has provided me with information, especially confirming what I am experiencing. The relationship between physical exercise, nutrition, and disease prevention was something I already knew because I had information. But it has confirmed and reaffirmed it for me. It motivates me to keep doing it.

#### 4.1.2 New information on health topics

The DSG sessions not only reinforced existing knowledge but also introduced new and relevant scientific information that participants had not previously encountered. This new knowledge expanded their understanding of health issues and enabled them to make connections with their personal and family experiences. The dialogic format of the gatherings allowed participants to collectively explore and assimilate this information, often leading to moments of discovery and shared learning.

Some of the participants stated that while they knew about the relationship between physical exercise, nutrition, and health conditions, they were unaware of the connection with cancer. This is the first intervention in which this statement was made:

It's true that generally we know that exercise and diet are important. But there are some things I didn't know, like when there's been a cancer diagnosis, for example, knowing that exercise can prevent it from recurring. Some details I didn't know. I think it's important to keep that in mind.

This reflection was echoed by others who expressed how the article had provided them with new insights: “I’ve gathered a lot of information that I did not have before, I’ve learned a lot. Honestly, I really liked it, especially what you can do to prevent cancer.”

In the DSG session on fibromyalgia, several interventions highlighted that they acquired new information about the wide range of symptoms and their relationship with other disorders that can complicate diagnosis. One participant emphasized this by reading a paragraph from the article’s introduction: “In the introduction it says: chronic pain, tender points, systemic symptoms, cognitive dysfunction, sleep disorder, anxiety, fatigue, depressive episodes. and it also says it’s associated with infections, psychiatric disorders, neurological disorders, diabetes, and rheumatic insufficiency”.

This new information resonated deeply with participants who had personal connections to the topic. For example, one woman related the article to her mother’s experience. The mother of this participant was diagnosed with fibromyalgia. She highlighted how, upon reading the article, she could identify symptoms associated with this disorder that her mother was experiencing but had not previously linked to fibromyalgia, such as disorientation. The information helped her better understand her mother’s situation, as the doctor had not explained this connection:

My mother often gets disoriented. She easily gets lost when she has to take a bus or in other situations. You have to keep calling her and saying, "Take this one and pay close attention." The doctor hadn't explained that this was also associated with fibromyalgia.

She also appreciated learning about the inflammatory component of the condition and how this new understanding could help her mother: “This is very valuable. I did not know this, and I believe my mother has never been told that it causes inflammation.” These exchanges illustrate how the DSGs served as a space for participants to connect scientific evidence with real-life situations, enhancing their understanding and sense of agency.

One of the new pieces of evidence that participants in the DSG on mental health and social bonds highlighted was the influence of emotional support from parents or caregivers during childhood as a preventive factor against depression in adulthood. However, receiving excessive protective support during childhood can lead to dependency, which instead of strengthening self-esteem, can weaken it and cause feelings of vulnerability. This was the first intervention on this evidence, followed by others who showed interest in this new knowledge about the impact of social support in childhood on self-esteem and vulnerability:

There is a section that states that the social support children receive can reinforce feelings of dependency as they grow older, weakening self-esteem and causing feelings of vulnerability. I understand that what is being conveyed here is the negative aspect of excessive support. It is positive to have social support, but when it is overly protective, it ultimately creates dependency.

The participants’ responses to this new information demonstrate their capacity to critically engage with scientific content and relate it to their own experiences and observations. The dialogic nature of the gatherings allowed for a deeper exploration of these insights, fostering a collective process of learning and reflection.

### 4.2 Interest in health-related topics

The DSG sessions not only facilitated knowledge acquisition but also generated a high level of interest and engagement among participants. This interest was rooted in the relevance of the topics—chosen by the women themselves—and was further amplified by the quality of the scientific texts and the dialogic format of the gatherings. The discussions revealed a strong motivation to learn, share, and reflect collectively, which contributed to a dynamic and inclusive learning environment.

#### 4.2.1 On the themes addressed in the articles

In the four DSG sessions, participants showed significant interest in the topics discussed. This was not only because these were subjects they were already interested in, having proposed them themselves, but also due to the quality of the texts they read and the way these topics were debated in the DSG.

One of the topics that generated the most interest in the DSG on fibromyalgia was the variety of non-pharmacological alternative treatments. Several participants expressed their surprise at the range of treatments available for fibromyalgia; one of these interventions was the following:

I had never heard anything like this before. There are so many treatments available. What I had heard was what you are saying: moderate exercise, swimming... I had never heard anything else, and look... (reading and pointing to a paragraph in the article) look... there are many treatments like acupuncture. Acupuncture is something that is more well-known, but I had never heard of it for fibromyalgia. I found it super interesting. There are things that, like you, I discovered today.

This sense of discovery was accompanied by a strong appreciation for the opportunity to share and reflect on the information collectively. Many of the participants’ interventions positively valued the DSG and the opportunity to share the knowledge provided in the articles. One of them expressed her satisfaction with the article and the chance to share this knowledge with her peers as follows: “I really liked the article and being here with you all today, sharing this has been very nice”.

The environment created in the DSG sessions, characterized by egalitarian dialog, respect for speaking turns, and appreciation of all opinions, generated great motivation among the participants. This setting allowed each person to feel heard and valued, fostering a richer exchange of ideas and deeper reflection on the topics discussed. The satisfaction expressed by these participants reflects how these elements contributed to an enriching and motivating experience for everyone involved.

In the DSG on social bonds and mental health, several interventions had already mentioned that the DSG sessions had helped increase interest and motivation in the topics by sharing their reflections with each other. They also noted that they paid attention to aspects they would not have considered if another participant had not highlighted them in her intervention. Three participants appreciated the alignment of thoughts among women from different areas and the reflection on new perspectives. One participant mentioned that “I found the gathering very interesting, especially what V said, that we are women from different areas, and we share very similar thoughts, and that motivates me.” Another added:

I did like the gathering. Talking about it among all of us makes me reflect because maybe I had focused on certain things. I had read it and paid attention to some aspects and not others. So, I was only going with what I thought about the article. It’s super interesting that through a text we read and discuss together, we learn other ways of seeing and reflecting on things that one hadn't noticed (when reading it alone before the debate).

A third participant commented that “Yes, regarding what R is saying, getting to know each other from different places, different profiles, with different aspirations and such, makes us have a more open mind.”

These reflections illustrate how the dialogic format of the DSGs fostered a space for mutual learning and the emergence of new perspectives through collective interpretation.

Mutual learning was also highlighted by the participants. The environment created in the DSG sessions, and the shared knowledge contribute to making it a space of support among women, as well as a space for mutual learning. Two of the participants highlighted this, mentioning the DSG as a space for mutual learning that contributes to the creation of a support network. One participant mentioned that “these spaces where we can share among all of us help in many other ways, not just to feel good or to have support but to learn from everyone.” Another participant elaborated:

Regarding what you and S were saying now, I understand that it also refers to how these networks not only help with mental health but also with everything you can learn. All the knowledge you can share in the discussion. I think that, at least for me, many times here (in the DSG), for example, with those who are here today, we see it a lot. Like maybe S knows something, explains it, and another woman who is connected, S, sees it and says, 'Oh, look,' and in the end, these networks and mutual help are created unintentionally, as sharing knowledge leads us to that.

The DSG sessions not only sparked significant interest among the participants regarding the topics discussed but also fostered an environment of support and mutual learning.

#### 4.2.2 On other health topics

The participants also expressed interest in other health topics that they connected with the themes of the articles they read. There were various interventions along these lines, particularly in the DSG on social ties and mental health. For instance, one of them highlighted the importance of social life in the prevention of Alzheimer’s: “I see the importance of socializing, of having a social life, even in preventing Alzheimer’s. Being mentally active and able to socialize seems beneficial. I believe it is very important, and we should encourage older people to interact more.”

This comment, along with others, reflects the participants’ proactive attitude toward health education and their desire to continue learning. The participants expressed their desire to continue learning more about these topics, emphasizing the importance of social life and mental activity in disease prevention and overall well-being. The DSGs thus served not only as a space for reflection on specific topics but also as a springboard for broader health-related curiosity and engagement.

### 4.3 Application of information

The DSG not only fostered knowledge acquisition and interest but also encouraged participants to apply the information discussed to their daily lives. The women reflected on how the scientific evidence could be integrated into their personal routines, family practices, and community engagement. These reflections revealed both the potential for behavioral change and the structural challenges that can hinder it. The dialogic format allowed participants to collectively explore strategies for applying what they learned, while also acknowledging the social conditions that shape their health decisions.

Several interventions in the DSG on physical activity, diet, and cancer were aimed at applying evidence to improve their health and well-being in their daily lives. However, this also led them to identify challenges such as the lack of social support that would facilitate adopting these changes. One of the participants mentioned that you can apply the evidence of exercising more and eating better to prevent diseases like cancer if you have social support that helps reduce the stress caused by the aforementioned socioeconomic difficulties; other participants made similar comments afterward:

Apart from stress, you get anxiety, and from there, I think you can even have a stroke with so much nervousness. Your brain can't take it anymore until it bursts. The best thing is to take it more calmly, but we can't because we don't have that support.

These interventions demonstrate that it is not only important to have information about the habits that can be adopted to improve health, but also to have the necessary social support to overcome challenging situations. The participants’ reflections show an awareness of the broader social determinants of health and the need for collective solutions.

Some of the interventions highlighted the importance of applying the evidence they read in this article to not only improve their children’s health but also to educate them in these habits. One participant mentioned how this information provided her with tools to educate her children and gave her peace of mind:

Above all, it gives you tools and also calms you down, because once you have the information, you can put it into practice. As a mother, it also gives you peace of mind regarding your children and how to educate them, especially thinking about them having a better life in the future than we did, which is super important.

This sentiment was echoed in the DSG on childhood obesity, where participants emphasized the value of discussing preventive guidelines together. The collective nature of the DSGs helped reinforce individual motivation and fostered a sense of shared responsibility. In this DSG, many particiopants made interventions aimed at applying the guidelines to prevent childhood obesity in their daily lives, reinforcing each other. One participant highlighted the importance of not only knowing these guidelines but also discussing them together in the DSG to gradually incorporate these habits into their daily routines: “It’s very good to have a discussion on this topic because that way we inform ourselves, talk about it, and become aware of what is available to put into practice”.

In the DSG on mental health and social ties, there were several interventions on how to apply evidence about the benefits of social support in their children’s emotional development. They emphasized the importance of the article providing evidence applicable to raising their children. One participant highlighted the significance of developing emotional intelligence in their children from an early age: “What I see here is the importance of emotional intelligence and managing emotions from a young age because it will influence them throughout their lives. That’s why it’s very important to teach this to our children”.

Another participant connected the importance of social and emotional support for their children’s development with having role models who act as guides during crucial moments in their lives: “I relate this, for example, to when children find a role model, someone who serves as a guide for them. Having that person there helps because, often, they have to make decisions where they might find themselves alone”.

These reflections illustrate how the DSGs enabled participants to translate scientific knowledge into practical parenting strategies, reinforcing their role as health educators within their families.

The participants also highly valued the contributions of the article on social ties and mental health. One of them highlighted its usefulness for a community project they were involved in with the women from their neighborhood: “If we had read this article for a project we had to do, it would have been very helpful because it talks about emotional well-being and mental health”.

This final example underscores how the DSGs not only supported individual learning and application but also contributed to collective initiatives aimed at improving community health. The sessions thus served as a bridge between scientific knowledge and real-world action, empowering participants to become agents of change in their own environments.

## 5 Discussion

The findings obtained from the four sessions of Dialogic Scientific Gatherings (DSG) with Roma women, most of whom do not have higher academic qualifications, reveal several key aspects about the impact of these gatherings in promoting an environment conducive to making informed decisions about health and well-being.

Firstly, the DSG sessions have proven effective in reaffirming the knowledge that participants already possessed. The Roma women identified and confirmed the relationship between stress, socioeconomic difficulties, and mental health, as well as the importance of social ties and community participation for emotional well-being. This finding is consistent with previous studies that emphasize the relevance of social support in mental health (6). The validation of their experiences through scientific evidence provided them with greater confidence to argue and apply this knowledge in their daily lives. This aspect is crucial, as the literature has highlighted that Roma women play a fundamental role in promoting health equity and reducing health disparities in their communities (1).

Additionally, the DSG sessions provided new relevant information that expanded the participants’ knowledge on health topics. For example, the relationship between physical activity and cancer prevention, as well as the wide range of symptoms associated with fibromyalgia, were novel aspects for many of them. This new knowledge not only allowed them to better understand their own health conditions and those of their family members, but also offered practical tools to improve their well-being. Acquiring new information is crucial for empowering Roma women to make informed decisions about their health (5).

The significant interest sparked by the DSG sessions among the participants regarding the topics discussed is another noteworthy aspect. The quality of the texts and the egalitarian debate format in the gatherings fostered an environment of mutual support and learning. The participants positively valued the opportunity to share and reflect on health topics, contributing to an enriching and motivating experience. This environment allowed each participant to feel heard and valued, promoting a richer exchange of ideas and deeper reflection on the topics addressed. This finding is consistent with the literature that highlights the importance of collaboration between Roma women, health organizations, researchers, and social services to address health inequalities (3).

The DSG sessions facilitated the application of information in the participants’ daily lives. The Roma women emphasized the importance of applying the evidence discussed in the gatherings to improve the health and well-being of their children, families, and themselves. The information provided gave them practical tools to educate their children on healthy habits and offered them peace of mind knowing they could use this knowledge for their families’ well-being. Additionally, identifying the challenges in adopting these changes, such as the lack of social support, underscores the need for a community environment that facilitates the implementation of healthy habits. This aspect is crucial, as the literature has highlighted that Roma women have led initiatives to preserve the health of their communities during the COVID-19 pandemic, challenging negative stereotypes about this group and emphasizing the importance of collaboration (3).

Although this case study and the DSGs were not designed to measure long-term health outcomes, the participants’ testimonies indicate increased awareness, motivation, and application of health knowledge in their daily lives. The dialogic methodology fostered inclusive participation, as evidenced by the diversity of voices and the egalitarian structure of the sessions. The mutual learning dynamic, where participants built on each other’s reflections, illustrates a form of assertive learning that empowered women to take ownership of health-related knowledge. This process of co-construction of meaning aligns with the principles of dialogic health communication, which emphasize horizontal, culturally responsive, and empowering interactions.

In this regard, the findings reinforce that DSGs exemplify a dialogic model of health communication that transcends the unidirectional transmission of information. Grounded in Freirean pedagogy and the theory of the dialogic society developed by Ramón Flecha, and drawing on the contributions of scholars such as Waisbord (14), Obregón and Waisbord (15), Schiavo (16), and Ganesh and Zoller (10), this model conceptualizes communication as a transformative process rooted in equity, social justice, and the co-creation of knowledge. DSGs operationalize this model by fostering spaces where scientific and experiential knowledge intersect, enabling participants—regardless of educational background—to engage in meaningful, egalitarian dialog that informs and empowers their health-related decision-making. Moreover, the DSGs reflect the principles of culturally sensitive and inclusive health risk communication, as advocated by Kreps (17) and Neuhauser and Kreps (18). The adaptation of scientific texts, the use of accessible language, and the recognition of participants’ lived experiences contributed to building trust and fostering engagement. The emphasis on egalitarian dialog and the prioritization of unheard voices further demonstrate how dialogic health communication can challenge traditional hierarchies and promote more equitable access to health knowledge.

The DSG sessions have proven to be an effective intervention for inclusive communication in promoting informed health decisions among non-academic Roma women. The reaffirmation of knowledge, acquisition of new information, significant interest in the topics discussed, and application of information in daily life are key aspects that highlight the positive impact of these gatherings. These elements contribute to empowering Roma women, improving their well-being, and reducing health inequalities in their communities. These findings shed light on future research regarding inclusive health communication in various contexts with vulnerable groups.

## Data Availability

The datasets are not directly available online to ensure the necessary level of confidentiality and the legitimate utilization of the data. Researchers interested in accessing any of the datasets are kindly requested to make a formal request by sending an email to AM-P amunte@ub.edu. This request should be accompanied by the following documents: a formal letter containing the researcher’s contact information, institutional affiliation, current position, the purpose of the research, details regarding the intended use of the data, and, if applicable, information about funding sources; an official letter from the researcher’s affiliated university or research institution confirming their association; and a confidentiality agreement, duly signed by the researcher, indicating their commitment to maintaining the confidentiality of the data.
